# Supplementary material for: Competition between inside-out unfolding and pathogenic aggregation in an amyloid-forming β-propeller
Source: Nat Commun. 2024 Jan 2;15:155. doi: 10.1038/s41467-023-44479-2 (PMC10762032; doi:10.1038/s41467-023-44479-2)
Supplement: Supplementary file 1 — Supplementary Information [file 41467_2023_44479_MOESM1_ESM.pdf]

Supplementary Information

Competition between inside-out unfolding and pathogenic aggregation in an amyloid-forming β-propeller

Emily G. Saccuzzo[1]*, Mubark D. Mebrat[2,3]*, Hailee F. Scelsi[1], Minjoo Kim[2,3], Minh Thu Ma[1], Xinya Su[4], Shannon E. Hill[1], Elisa Rheaume[5], Renhao Li[6,7], Matthew P. Torres[4], James C. Gumbart[1,4,7], Wade D. Van Horn[2,3], Raquel L. Lieberman[1]

[1]School of Chemistry & Biochemistry, Georgia Institute of Technology

[2]Biodesign Center for Personalized Diagnostics, Arizona State University

[3]School of Molecular Sciences, Arizona State University

[4]School of Biological Sciences, Georgia Institute of Technology

[5]Interdisciplinary Graduate Program in Quantitative Biosciences, Georgia Institute of Technology

[6]Aflac Cancer and Blood Disorders Center, Children's Healthcare of Atlanta and Department of Pediatrics, Emory University School of Medicine

[7]School of Physics, Georgia Institute of Technology

*These authors contributed equally

Correspondence: Wade D. Van Horn wade.van.horn@asu.edu or Raquel L. Lieberman Raquel.lieberman@chemistry.gatech.edu

Contents:

Supplementary Figure 1-14

Supplementary Tables 1-8

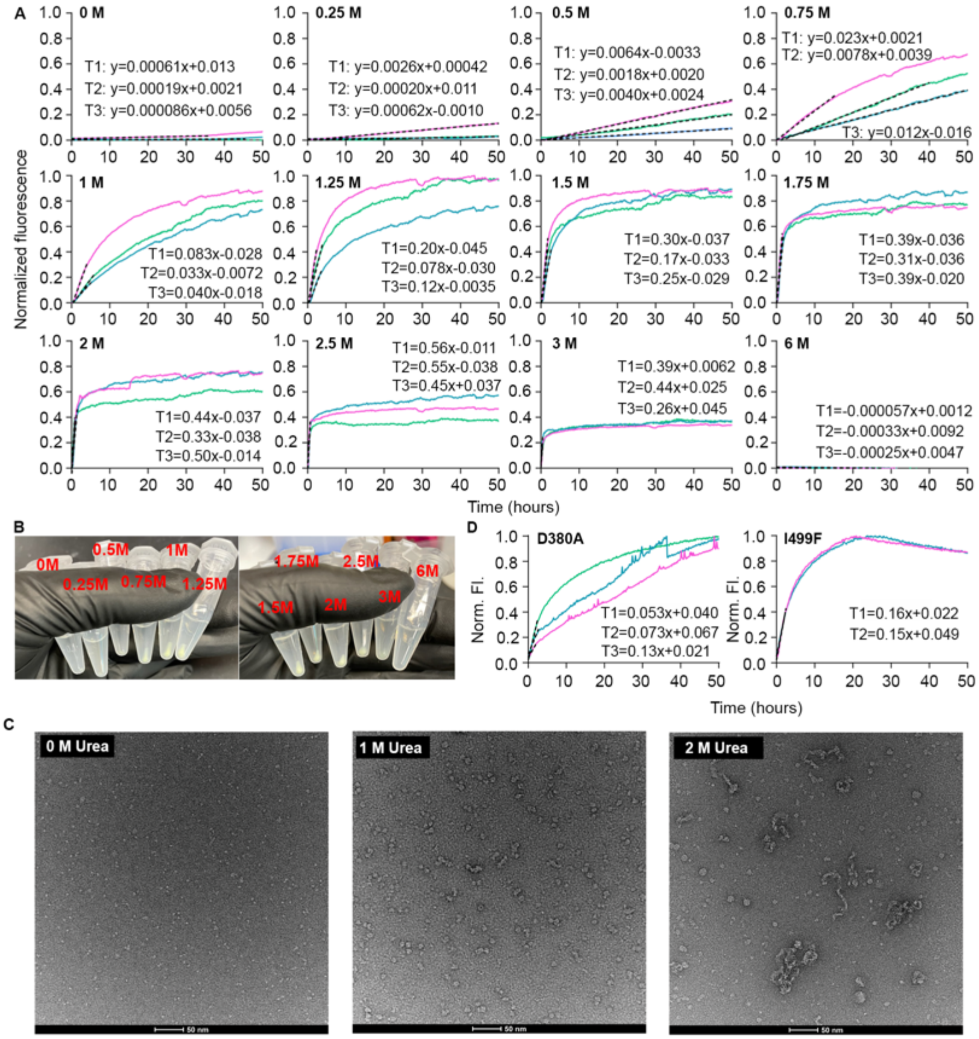

Supplementary Figure 1. Aggregation of urea-unfolded OLF^{WT} and as-purified disease mutants. (A) OLF^{WT} fibril formation at 37 °C as a function of urea concentration. ThT fluorescence was monitored over time. Each trace represents the average of technical duplicates for three independently purified OLF preparations. Initial rate was evaluated using linear regression, represented as a black dotted line for each trace. The corresponding equation is labeled on each graph. Trial 1, pink; Trial 2, blue; Trial 3, green. Source data are provided as a Source Data file. (B) Pellets of representative samples from (A) after termination of experiment. Yellow-tinged pellets were visible for samples incubated with 0.25-3 M urea. (C) ThT aggregation kinetics for disease variants D380A (red) and I499F (green) replotted from[1]. Initial rate and colors evaluated as for (A). Source data are provided as a Source Data file. (D) Negative stain TEM images of end-point aggregates formed by urea-induced OLF^{WT}. Representative samples from Figure 2A/ Supplementary Fig. 1A were deposited onto negatively glow-discharged carbon-coated copper grids and stained with 0.7% uranyl formate. Samples were imaged on an FEI 200 kV Talos Arctica equipped with a K3 detector. At 0 M urea (left), particles ~4 nm in diameter are observed, the expected size of monomer. At 1 M (middle), donut shaped particles with diameter of ~7 nm are apparent, consistent with an oligomeric species. At 2.5 M urea (right), a variety of fibrillar species are observed. Imaging conducted once.

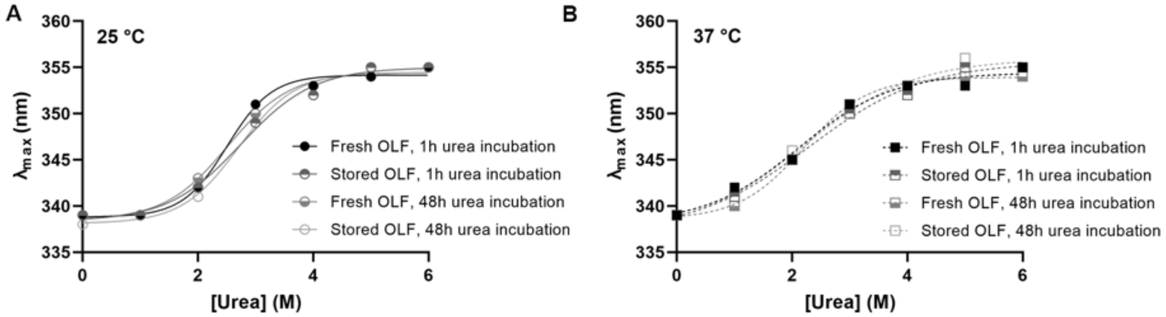

Supplementary Figure 2. Unfolding of OLF with short and long incubation times in urea with fresh versus stored protein are indistinguishable. (A) comparison of data acquired at 25 °C (B) comparison of data acquired at 37 °C. Data are presented as the average of analytical triplicates. Source data are provided as a Source Data file.

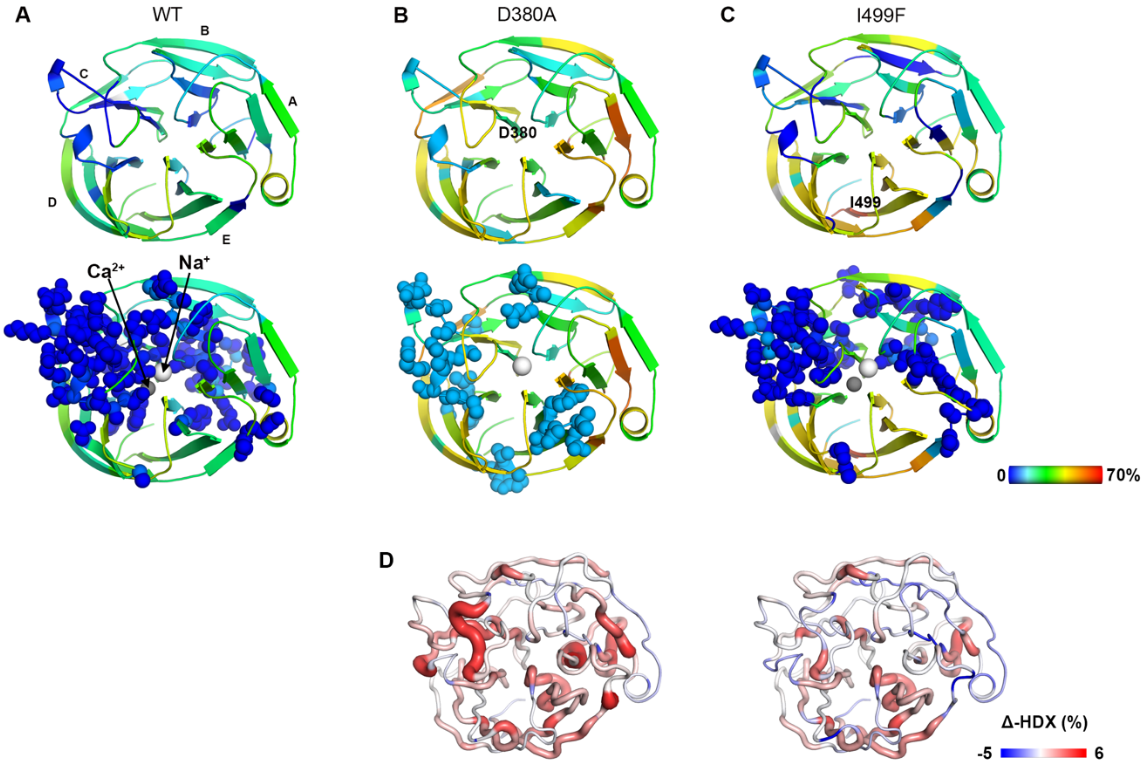

Supplementary Figure 3. Comparison of HDX-MS maximal uptake (10,000s) for WT, D380A, and I499F mapped onto the OLF^{WT} structure. (A-C) Top: ribbon diagram. Bottom: ribbon diagram with regions of least overall exchange highlighted. Extent of exchange indicated by rainbow scale bar from 0-70%. (D) Difference in maximal (10,000s) uptake compared to OLF^{WT} for OLF^{D380A} and OLF^{I499F}. Extent of difference (%)are indicated by blue (negative) to red (positive), with white indicating no difference. Overall, OLF^{D380A} experiences higher levels of exchange compared to OLF^{WT}, whereas OLF^{I499F} is more variable but some regions appear to be the same or less than OLF^{WT}. See also Supplementary Figure 4, Supplementary Figure 5.

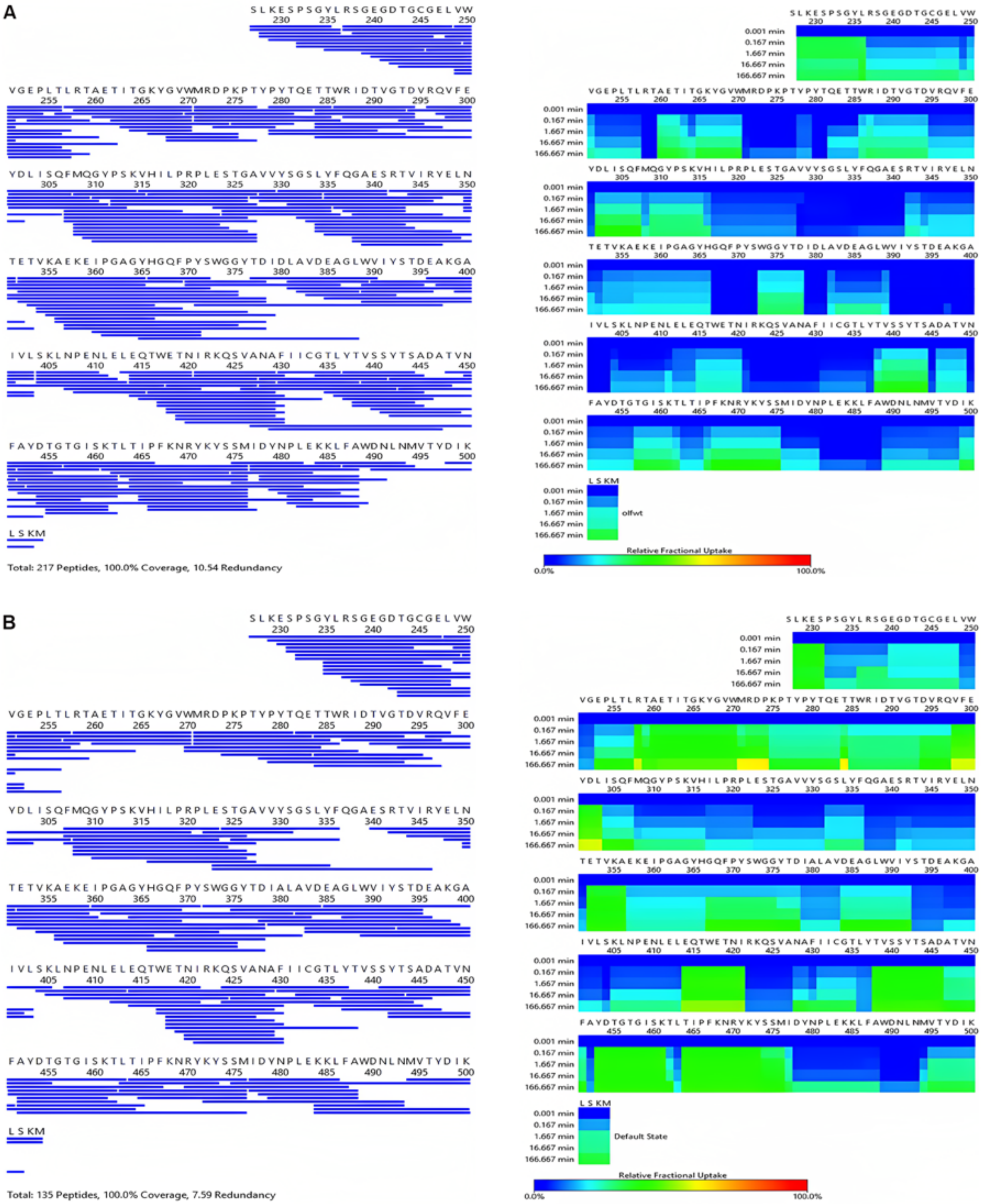
A
Total: 217 Peptides, 100.0% Coverage, 10.54 Redundancy
0.001 min
0.167 min
1.667 min
16.667 min
166.667 min
olfwt
Relative Fractional Uptake
0.0%
100.0%
B
Total: 135 Peptides, 100.0% Coverage, 7.59 Redundancy
Default State
Relative Fractional Uptake
0.0%
100.0%

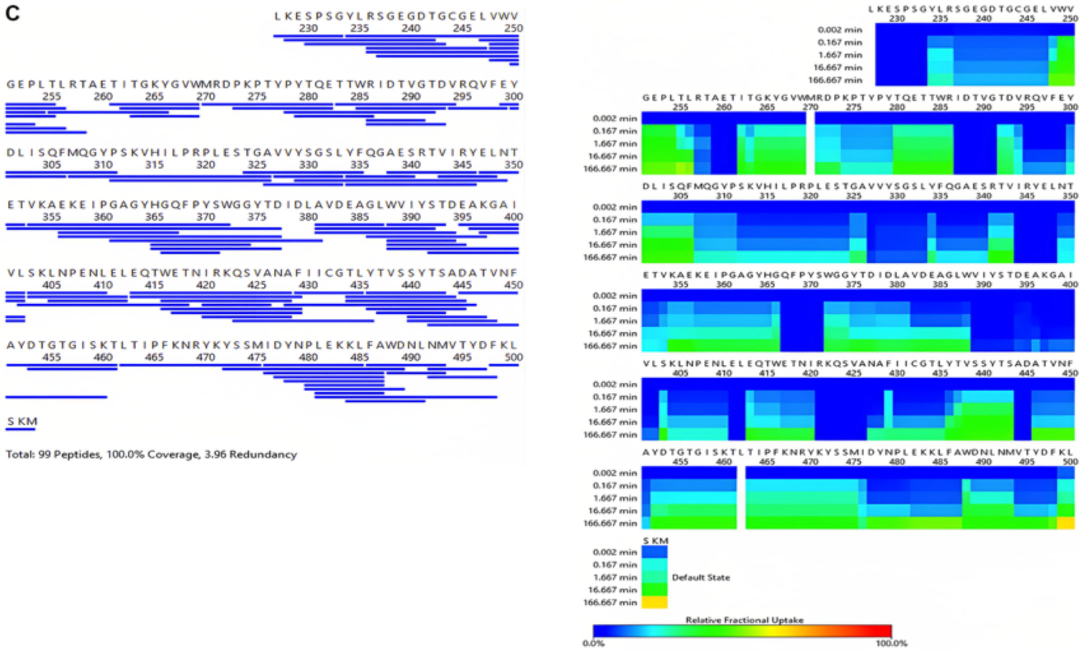

Supplementary Figure 4. Summary of HDX-MS coverage (left) and heat map (right) data for (A) OLF^{WT}, (B) OLF^{D380A}, and (C) OLF^{I499F}. Left: each blue bar represents a peptide fragment reproducibly detected after HDX-MS labeling reactions. Right: Heat map showing levels of deuterium uptake for each labeling time point. Relative fractional uptake colored blue to red (0-100%) as indicated in the scale bar. See also Supplementary Figure 3, Supplementary Figure 5.

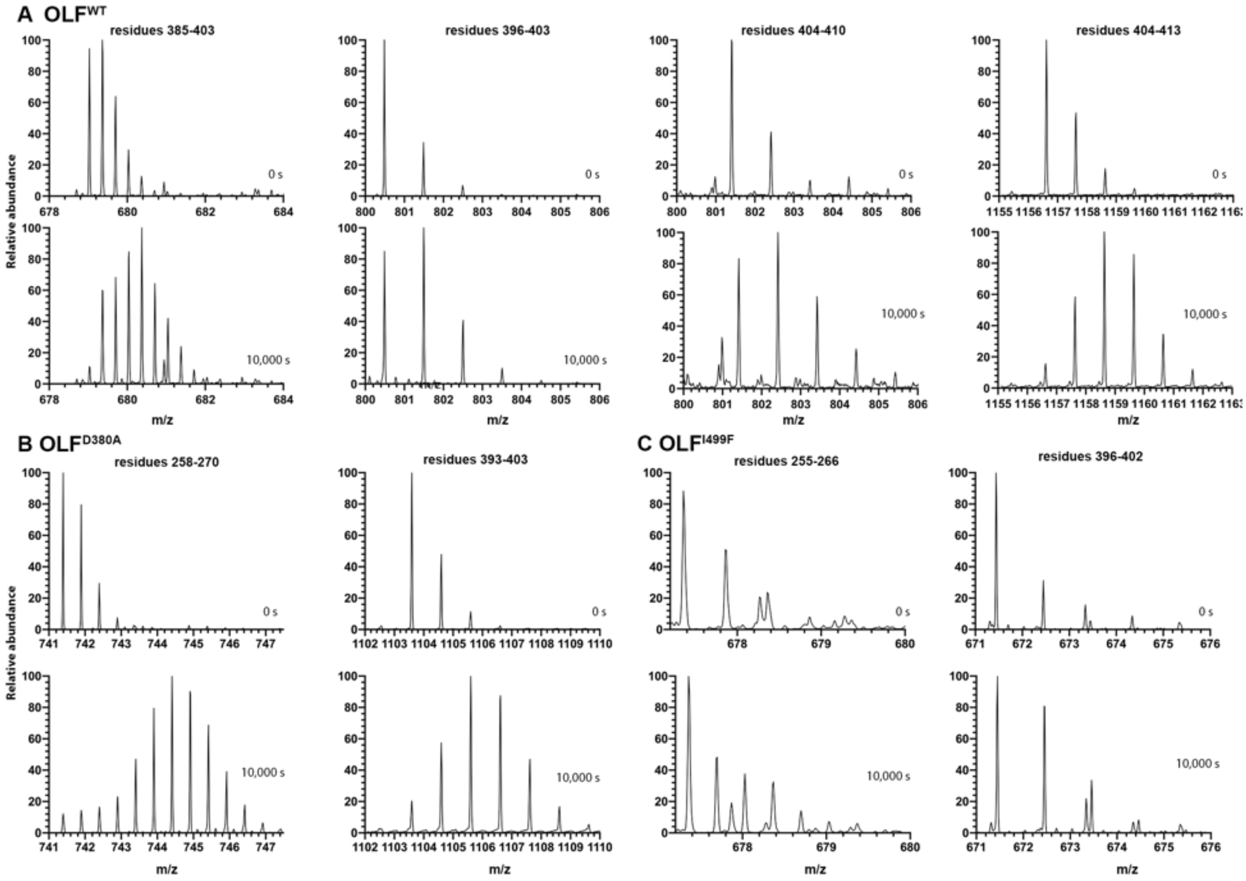

Supplementary Figure 5. Representative mass spectra for selective peptides after 0 and 10,000-second incubation. (A) OLF^{WT}. (B) OLF^{D380A}. (C) OLF^{I499F}. See also Supplementary Figure 3, Supplementary Figure S4.

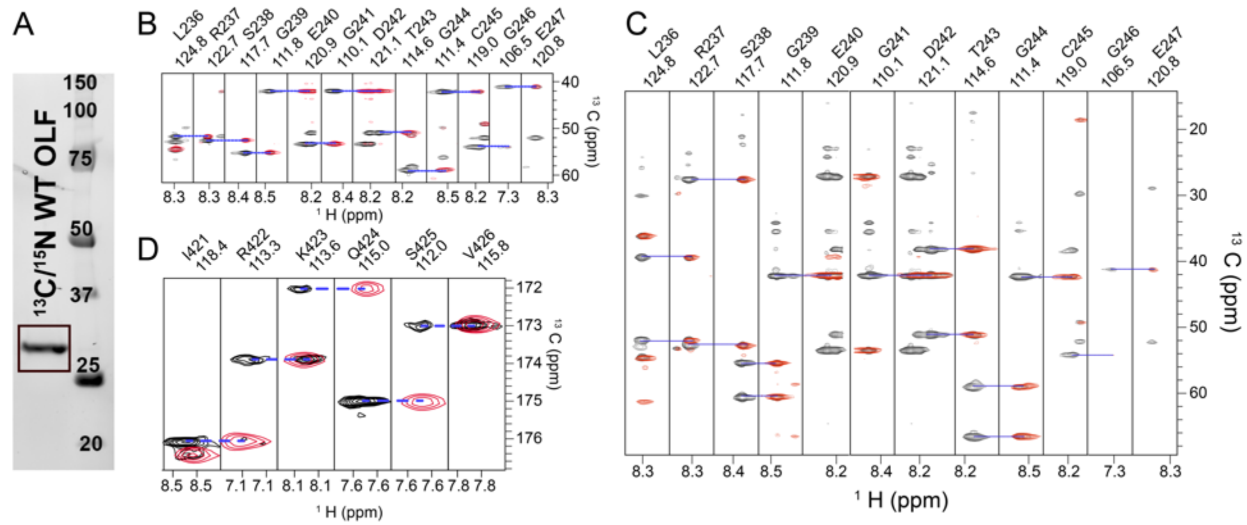

Supplementary Figure 6. Representative solution NMR data used in OLF amide backbone resonance assignment. (A) 12% SDS-PAGE of pure $^{13}C/^{15}N$ WT OLF sample used for 3D NMR experiments. Ladder in kDa. See also Supplementary Figure 14. (B) Strip plot representation of TROSY-based 3D HNCA (black)/HNcoCA (red) data from residues Leu236–Glu247 in the OLF N-terminus. (C) Equivalent 3D HNCACB (black)/HNcoCACB (red) data for the same region as panel B. (D) A strip plot of HNCO (red)/HNcaCO (black) from residues located spatially near D380. For B-D, blue lines connect 3D NMR strips from adjacent residues (i-1 to i) along the OLF backbone. OLF backbone assignment data has been deposited to BMRB 51750 [https://dx.doi.org/10.13018/BMR51750]. Source data are provided as a Source Data file.

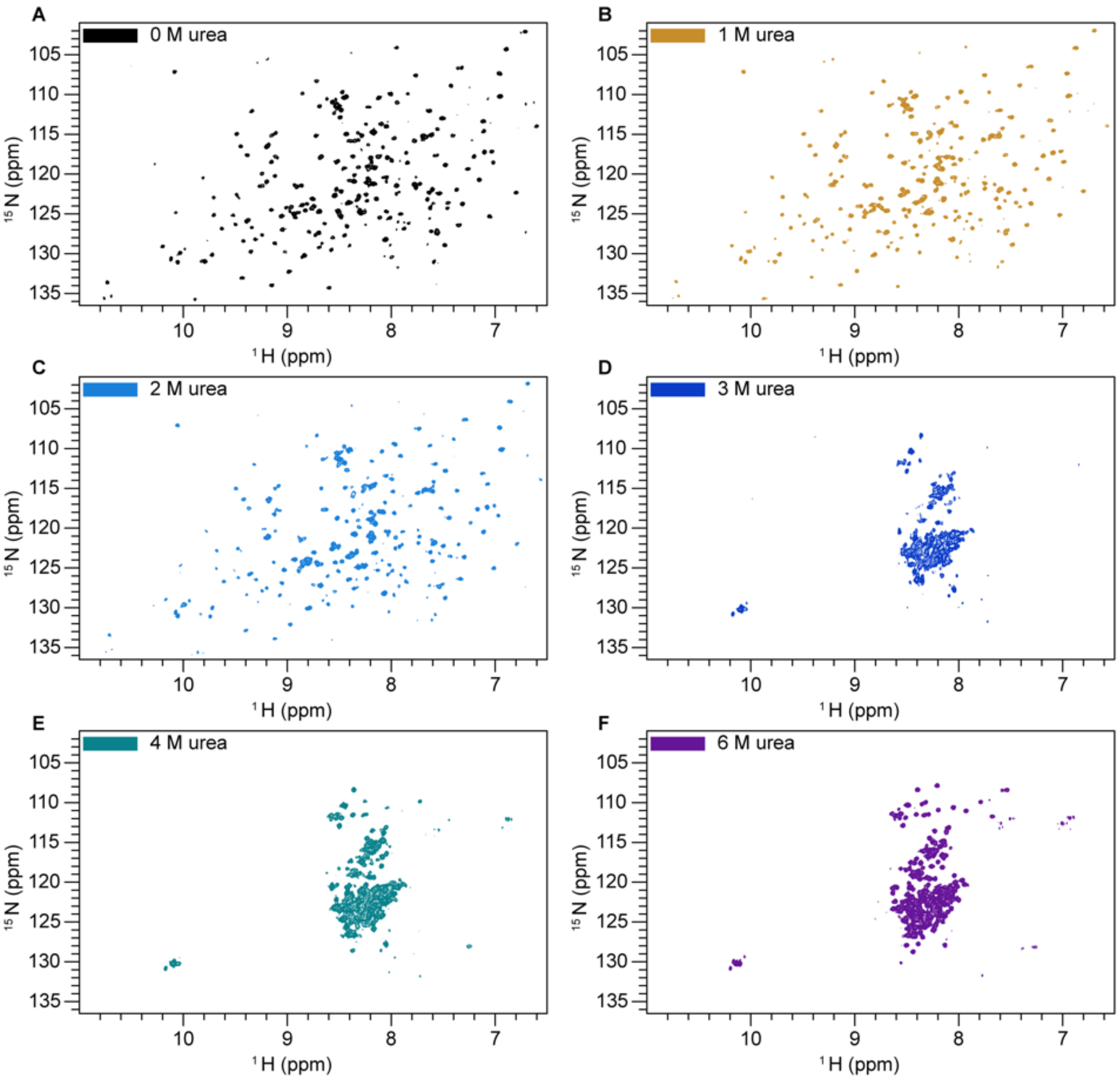

Supplementary Figure 7. OLF^{WT} urea-based chemical unfolding. Individual ^{1}H-^{15}N TROSY-HSQC spectra of OLF^{WT} in the presence of 0 M urea (A, black), 1 M urea (B, gold), 2 M urea (C, light blue), 3 M urea (D, dark blue), 4 M urea (E, teal), 6 M urea (F, purple) are shown. These data are overlayed in Figure 2A.

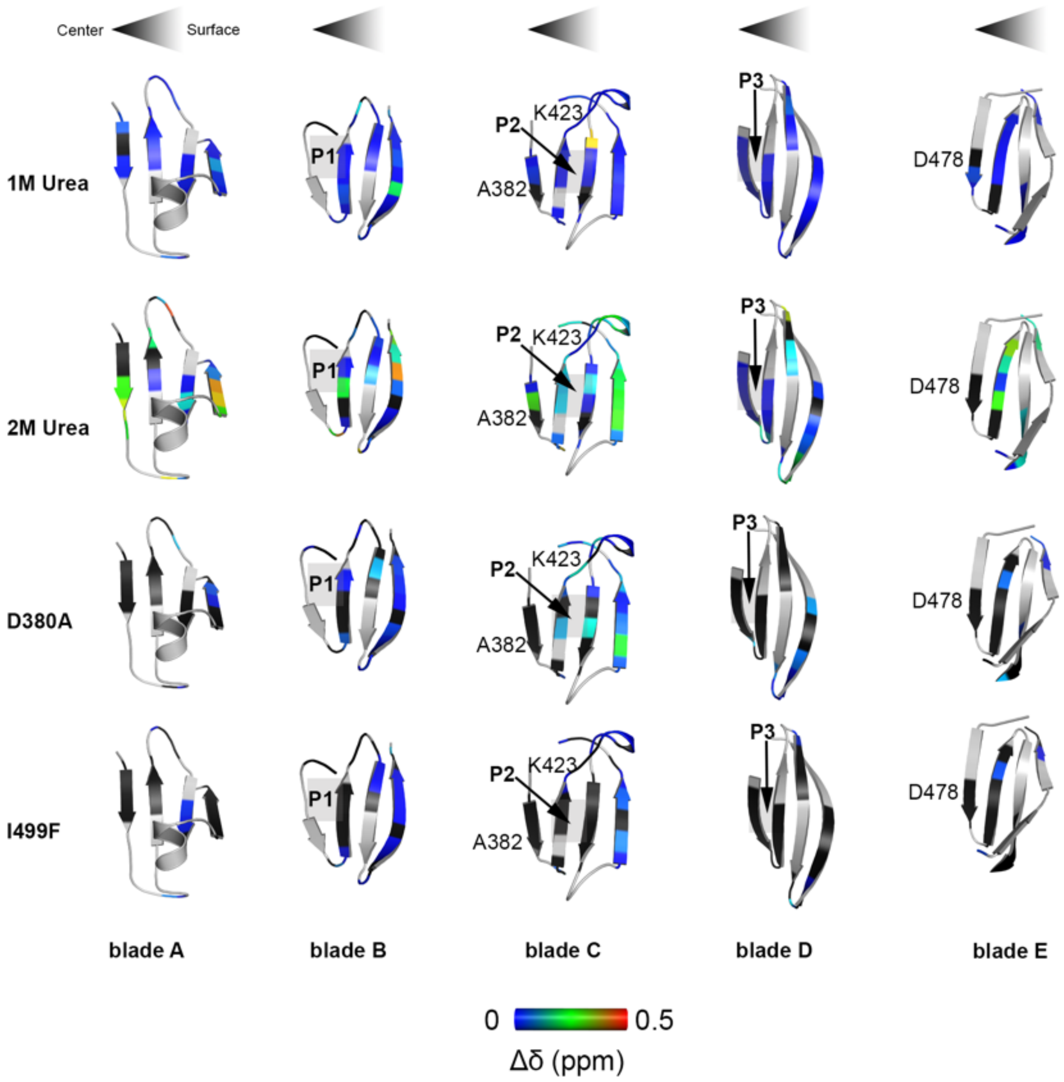

Supplementary Figure 8. Blade-by-blade comparison of chemical shift perturbation (CSP) for OLF^{WT} in 1M urea, WT OLF in 2M urea, and disease variants D380A and I499F. Residues on the interior near the metal binding center, eg. A382 and D478, are no longer present, whereas across all four samples the exterior strands, e.g. of blades B and C, remain intact. CSP is colored from blue (0 Δδ) to red (0.5 Δδ) for increasing resonance position changes. Regions of the structure that are colored gray are not assigned and those colored black are missing relative to the OLF^{WT} spectrum. Source data are provided as a Source Data file.

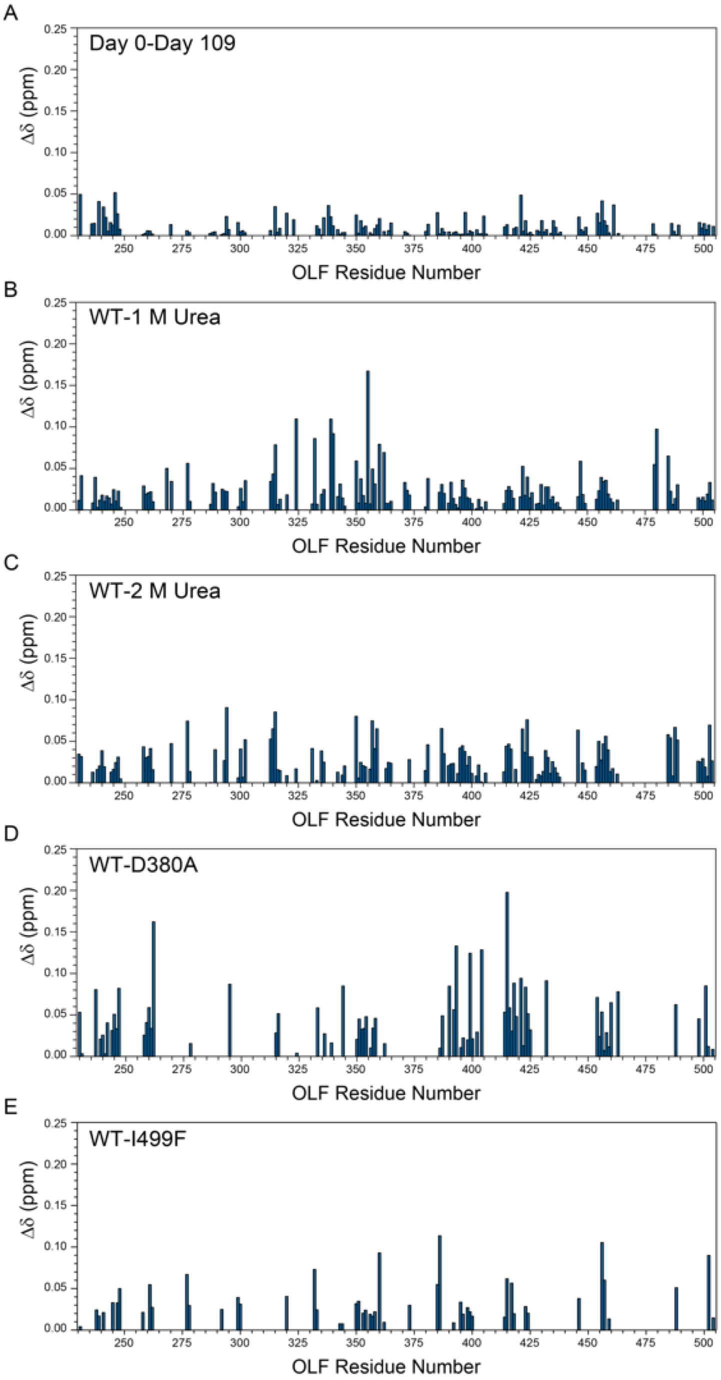

Supplementary Figure 9. OLF ^{1}H-^{15}N HSQC chemical shift changes (Δδ) analysis. Chemical shift changes (A) after 109 days, in the presence of (B) 1 M Urea, (C) 2 M urea and in the mutants (D) D380A and (E) I499F. Source data are provided as a Source Data file.

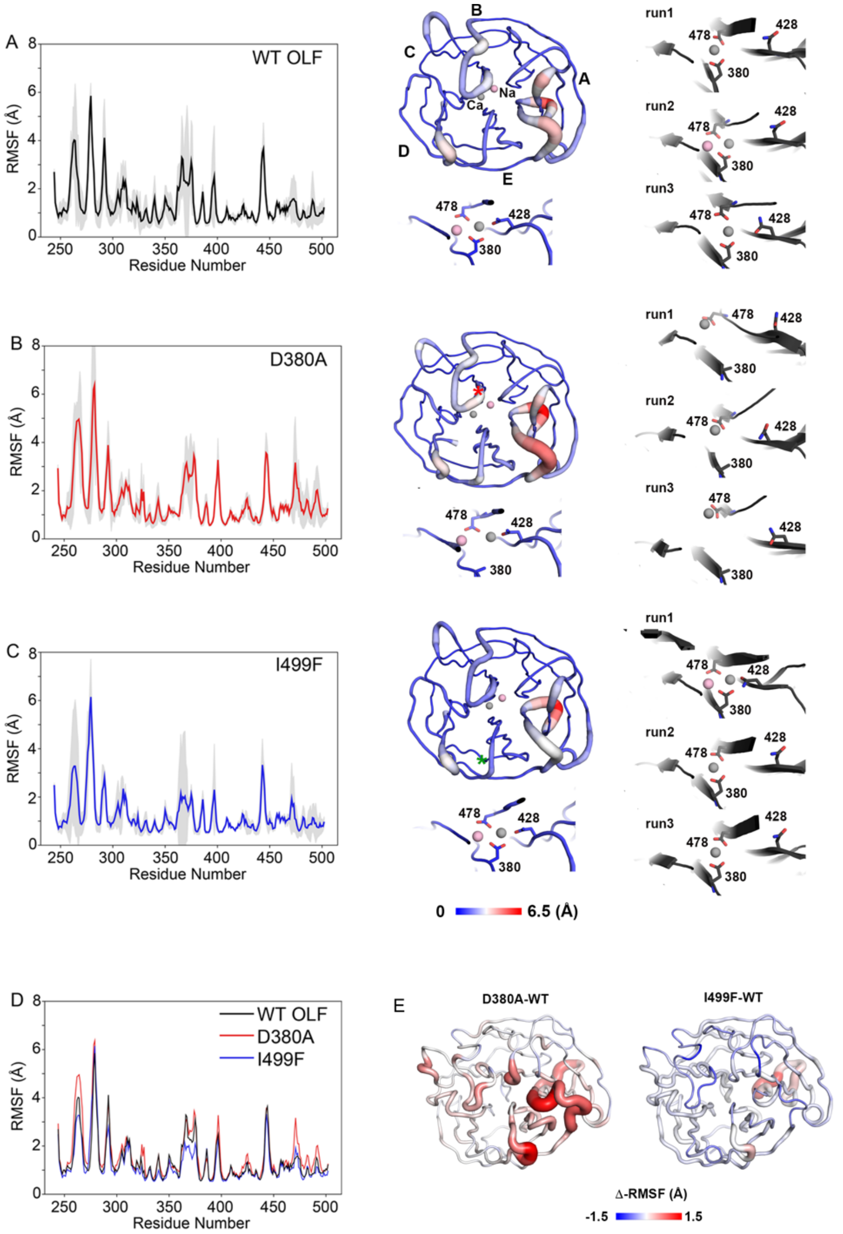

Supplementary Figure 10. MD simulations plotted per-residue and mapped onto the OLF structure. (A) OLF^{WT} (black), (B), OLF^{D380A} (red), (C) OLF^{I499F} (blue). Left, RMSF vs residue number curves represent the average of three 1-µs simulations at 293 K. The grey shading above and below represent +/-1 SD, respectively. Middle, OLF structure after initial equilibration, with RMSF values mapped on structure with sausage ribbon representation and color in range 0 (blue) -6.5 Å (red). Right, final structure of metal centers for each of three runs. For structures in (A-C), RMSF values are indicated in a range from blue (0 Å) to red (6.5 Å), with white being an intermediate RMSF. (D) Overlay of RMSF versus residue number curves for OLF^{WT}, OLF^{D380A} and OLF^{I499F}. Colors of traces as in A-C. (E) Difference in average RMSF between each mutant and WT, mapped onto the structure. Source data are provided as a Source Data file.

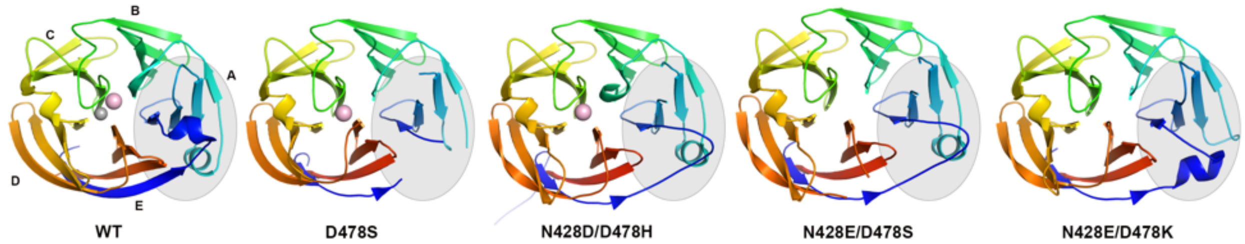

Supplementary Figure 11. Comparison of WT and structurally characterized *de novo* OLF variants with partial misfolding. Original wild-type OLF (4WXQ, left), and D478 single and double point variants as indicated, showing modifications in blade A (grey oval) when metal center ligand(s) are mutated. Grey sphere, Ca^{2+}, pink sphere, Na^{+}. Structures reproduced from[2-4]. Ribbon diagrams colored in rainbow from N terminus (blue) to C terminus (red).

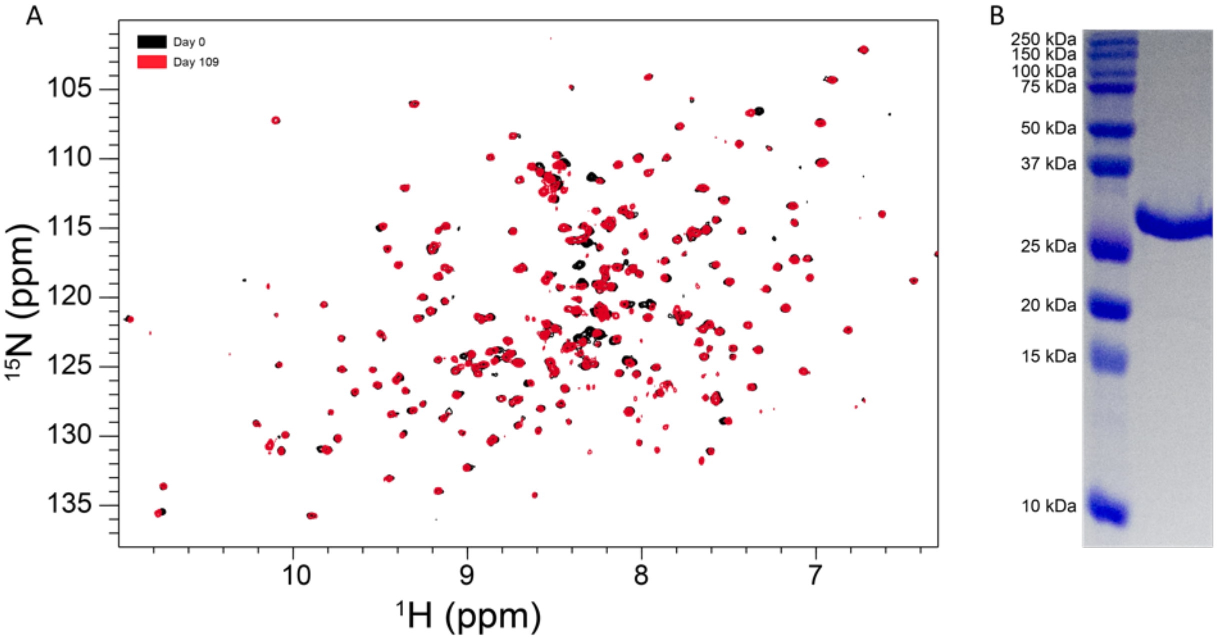

Supplementary Figure 12. Months-long ^{1}H-^{15}N TROSY-HSQC spectra of OLF^{WT}. (A) Initial spectrum (Black) is overlaid with one collected from the same sample stored at room temperature after 109 days (red). (B) 15% SDS-PAGE Coomassie-stained gel of the OLF^{WT} NMR sample after the long-timescale NMR kinetics experiment (>109 days) reached equilibrium. The gel shows no evidence of proteolysis. See also Supplementary Figure 14.

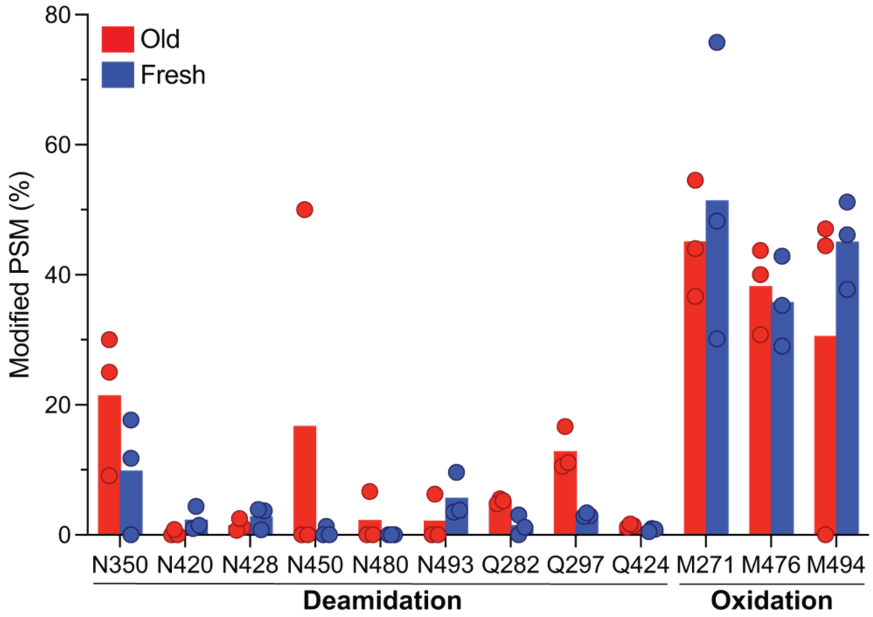

Supplementary Figure 13. Comparison of methionine oxidation and deamidation between freshly purified OLF^{WT} and NMR sample used for analysis in Figure 7 after 1 year storage at 4 °C. Data are means from 3 technical replicates, individual data points indicated with circles. Old sample, red. Fresh sample, blue. Source data are provided as a Source Data file.

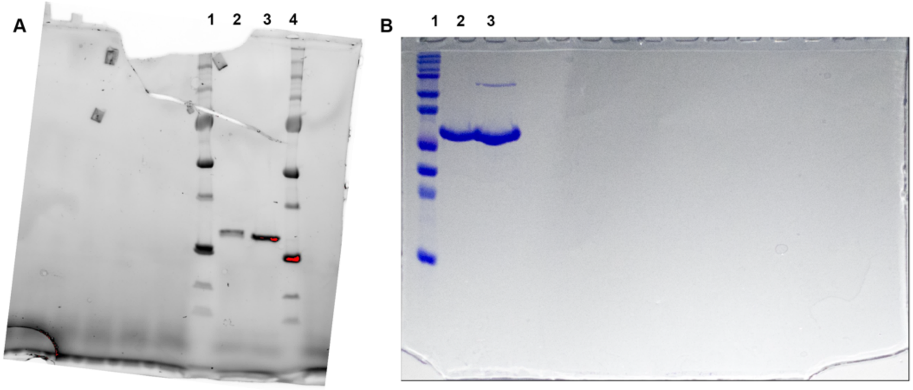

3/07/2022 12% SDS-PAGE
Lane 1: Ladder
Lane 2: As-purified $^{13}C/^{15}N$ OLF^{WT} 5x diluted
Lane 3: As-purified $^{13}C/^{15}N$ OLF^{WT} not diluted
Lane 4: Ladder

9/28/2022 15% SDS-PAGE
Lane 1 Ladder
Lane 2 OLF^{WT} Used for long timescale dynamics
Lane 3 OLF^{WT} Used for backbone assignment

Supplementary Figure 14. Uncropped gels. (A) Supplementary Figure 6A. (B) Supplementary Figure 12B.

Supplementary Table 1. Equations and R-squared values for lines of best fit for all WT OLF/urea conditions.

Urea Concentration		Trial 1	Trial 2	Trial 3
0 M	Equation	y=0.00061x+0.013	y=0.00019x+0.021	y=0.000068x+0.0056
	R-squared	0.970	0.566	0.227
0.25 M	Equation	y=0.0026x+0.00042	y=0.00020x+0.011	y=0.00062x-0.0010
	R-squared	0.998	0.803	0.848
0.5 M	Equation	y=0.0064x-0.0033	y=0.0018x+0.0020	y=0.0040x+0.0024
	R-squared	0.998	0.987	0.987
0.75 M	Equation	y=0.023x+0.0021	y+0.0078x+0.0039	y=0.012x-0.016
	R-squared	0.993	0.999	0.998
1 M	Equation	y=0.083x-0.028	y=0.033x-0.0072	y=0.040x-0.018
	R-squared	0.989	0.995	0.996
1.25 M	Equation	y=0.20x-0.045	0.078x-0.030	y=0.12x-0.0035
	R-squared	0.989	0.983	0.982
1.5 M	Equation	y=0.30x-0.037	y=0.17x-0.033	y=0.25x-0.029
	R-squared	0.973	0.980	0.964
1.75 M	Equation	y=0.39x-0.036	y=0.31x-0.036	y=0.39x-0.020
	R-squared	0.966	0.965	0.958
2 M	Equation	y=0.44x-0.037	y=0.33x-0.038	y=0.50x-0.014
	R-squared	0.961	0.961	0.957
2.5 M	Equation	y=0.56x-0.011	y=0.54x-0.038	y=0.45x+0.037
	R-squared	0.956	0.951	0.984
3 M	Equation	y=0.39x-0.025	y=0.44x+0.025	y=0.26x+0.045
	R-squared	0.965	0.975	0.995
6 M	Equation	y=-0.000057x+0.0012	y=-0.00033x+0.0092	y=-0.00025x+0.0047
	R-squared	0.531	0.968	0.576
D380A	Equation	y=0.053x+.040	y=0.073x+0.067	y=0.13x+0.021
	R-squared	0.988	0.980	0.987
I499F	Equation	y=0.16x+0.022	y=0.15x+0.049	[redacted]
	R-squared	0.985	0.996	

Supplementary Table 2. Metal analysis from ICP-OES.

Sample	Ca^{2+}:OLF (per mol)
WT	0.73
I499F	0.17

Supplementary Table 3. HDX Data Summary

Data Set	OLF^{WT}	OLF^{D380A}	OLF^{I499F}
HDX reaction details[a]	10 mM phosphate buffer, 99.9% D_2O, pD 7.0, 20 °C		
HDX time course	0.06s (0.001m), 10s (0.167m), 100s (1.667m), 1000s (16.67m), 10000s (166.667m)		
HDX control samples	Undeuterated controls	Undeuterated controls	Undeuterated controls
Back-exchange (mean / IQR)	N/A[b]		
# of Peptides	217	135	99
Sequence coverage	100%	100%	100%
Redundancy	10.54	7.59	3.96
Replicates	3 (technical), 5 repeats per time point		
Repeatability	± 0.2 relative Da		
Significant differences in HDX (ΔHDX > X D)	> 0.50 Da		

[a] 1:7 (v:v) dilution with D_2O-containing buffer
1:1 (v:v) dilution with quench buffer [100 mM phosphate, 0.5 M tris(2-carboxyethyl)phosphine, 0.8% formic acid, 2% acetonitrile, pH 2.5]
[b] not measured in these experiments; 30-45% estimated based on other experiments conducted on same instrument in similar time frame

Supplementary Table 4. Primary principal component values of NMR-detected OLF urea titration.

[Urea]	PC1
0	0.0567
1	0.1498
2	0.2893
3	0.6428
4	0.7637
6	0.987

Supplementary Table 5. Data collection and refinement statistics (molecular replacement)

	OLF^{WT}
**Data collection**	
Space group	P 1 2_1 1
Cell dimensions	
a, *b*, *c* (Å)	49.47 50.53 50.87
α, β, γ (□)	90, 96.25, 90
Resolution (Å)	27.91 - 1.27 (1.32 - 1.27) *
R_{merge}	0.08769 (0.5015)
I / σ*I*	16.41 (2.89)
Completeness (%)	95.26 (91.44)
Redundancy	6.4 (5.3)
**Refinement**	
Resolution (Å)	27.91 - 1.27 (1.315 - 1.27)
No. reflections	62697 (5947)
R_{work} / R_{free}	0.140 (0.184) / 0.156 (0.209)
No. atoms	
Protein	2254
Ligand/ion	16
Water	282
B-factors	
Protein	15.11
Ligand/ion	23.54
Water	29.12
R.m.s. deviations	
Bond lengths (Å)	0.020
Bond angles (□)	1.59

*Single crystal used for structure determination. Values in parentheses are for highest-resolution shell.

Supplementary Table 6. Primary principal component values of months-long NMR-detected kinetics.

Day	PC1
0	1
7	0.71
33	0.55
42	0.28
51	0.19
56	0.14
67	0
79	0.05
90	0.06
105	0.05
109	0.06

Supplementary Table 7. NMR experiment parameters for OLF^{WT} backbone assignment

Expt.	Pulse Program	NS	F1 (direct)			F2 (indirect)			F3 (indirect)			NUS (%)	NUS T2
			TD	SW (ppm)	O1p (ppm)	TD	SW (ppm)	O2p (ppm)	TD	SW (ppm)	O3p (ppm)		
^{15}N, ^{1}H HSQC	*trosyetf3gpsi*	4	2k	12.97	4.696	128	42.5	118	----	--------	------	------	------
HNCA	*trhncaetgp3d*	56	2k	12.97	4.696	64	42.5	118	96	30	53.2	46	0.006
HNCA	*trhncaetgp3d*	72	2k	12.97	4.696	64	42.5	118	96	30	53.2	46	0.006
HN(CO)CA	*trhncocaetgp3d*	16	2k	12.97	4.696	64	42.5	118	96	30	53.2	32	0.008
HN(CO)CA	*trhncocaetgp3d*	48	2k	12.97	4.696	64	42.5	118	96	30	53.2	32	0.004/6
HN(CO)CA	*trhncocaetgp3d*	72	2k	12.97	4.696	64	42.5	118	96	30	53.2	40	0.004/6
HNCACB	*trhncacbetgp3d*	48	2k	12.97	4.696	64	42.5	118	96	75	39	35	0.007
HNCACB	*trhncacbetgp3d*	64	2k	12.97	4.696	64	42.5	118	96	75	39	35	0.007
HNCACB	*trhncacbetgp3d*	128	2k	12.97	4.696	64	42.5	118	96	75	39	35	0.007
CBCA(CO)NH	*trcbcaconhetgp3d*	64	2k	12.97	4.699	64	42.5	118	96	75	39	45	0.007
CBCA(CO)NH	*trcbcaconhetgp3d*	96	2k	12.97	4.696	64	42.5	118	96	75	39	52	0.004/6
HNCO	*trhncoetgp3d*	40	2k	12.97	4.696	64	42.5	118	96	16	172	52	0.004/6
HNCO	*trhncoetgp3d*	40	2k	12.97	4.696	64	42.5	118	96	16	172	52	0.004/6
HN(CA)CO	*trhncacoetgp3d*	40	2k	12.97	4.699	64	42.5	118	80	16	172	48	0.004/6

-All experiments were collected using $^{1}H/^{15}N/^{13}C$ labeled WT-OLF (570 µM) in 3 mm NMR tube at 25 °C (298 K), with 2.2% D_2O
-Buffer: 10 mM KH_2PO_4, 10 mM Na_2HPO_4, 200 mM NaCl, pH 6.8
-Instrument: Bruker 850 MHz ^{1}H spectrometer and Avance III HD console equipped with 5 mm TCI Cryoprobe
-Pulse programs: All NMR pulse programs were used unmodified from the Bruker Topspin pulse program library.
-Abbreviations used: NS, number of transients; TD, number of points; SW, spectral width; O#p, carrier frequency; NUS, non-uniformed sampling

Supplementary Table 8. NMR experiment parameters for Urea titration of OLF^{WT}, OLF^{D380A}, OLF^{I499F}, and OLF^{WT} long-timescale dynamics

OLF sample	Expt.	Pulse Program	NS	F1 (direct)			F2 (indirect)		
				TD	SW (ppm)	O1p (ppm)	TD	SW (ppm)	O2p (ppm)
Urea titration	^{15}N, ^{1}H HSQC	*trosyeft3gpsi*	128	2k	12.97	4.696	128	42.5	118
D380A	^{15}N, ^{1}H HSQC	*trosyeft3gpsi*	280	2k	12.97	4.696	128	42.5	118
I499F	^{15}N, ^{1}H HSQC	*trosyeft3gpsi*	380	2k	12.97	4.696	128	42.5	118
Long time scale dynamics	^{15}N, ^{1}H HSQC	*trosyeft3gpsi*	64	2k	12.97	4.696	128	42.5	118

-All experiment were collected using $^{1}H/^{15}N$ labeled WT-OLF, D380A-OLF, or I499F-OLF in 3 mm NMR tube at 25 °C (298 K) in ~2.7% D_2O.
-Buffer: 10 mM KH_2PO_4, 10 mM Na_2HPO_4, 200 mM NaCl, pH 6.8
-Instrument: Bruker 850 MHz ^{1}H spectrometer and Avance III HD console equipped with 5 mm TCI Cryoprobe
-Pulse programs: All NMR pulse programs were used unmodified from the Bruker Topspin pulse program library.
-Abbreviations: NS, number of transients; TD, number of points; SW, spectral width; O#p, carrier frequency

Supplementary References

1 Hill, S. E., Donegan, R. K. & Lieberman, R. L. The glaucoma-associated olfactomedin domain of myocilin forms polymorphic fibrils that are constrained by partial unfolding and peptide sequence. *J Mol Biol* **426**, 921-935 (2014).

2 Donegan, R. K. *et al.* Structural basis for misfolding in myocilin-associated glaucoma. *Hum Mol Genet* **24**, 2111-2124 (2015).

3 Hill, S. E., Cho, H., Raut, P. & Lieberman, R. L. Calcium-ligand variants of the myocilin olfactomedin propeller selected from invertebrate phyla reveal cross-talk with N-terminal blade and surface helices. *Acta Crystallogr D Biol Crystallogr* **75**, 817-824 (2019).

4 Hill, S. E. *et al.* Stable calcium-free myocilin olfactomedin domain variants reveal challenges in differentiating between benign and glaucoma-causing mutations. *J Biol Chem* **294**, 12717-12728 (2019).
