# Supplementary material for: Competition between inside-out unfolding and pathogenic aggregation in an amyloid-forming β-propeller
Source: Nat Commun. 2024 Jan 2;15:155. doi: 10.1038/s41467-023-44479-2 (PMC10762032; doi:10.1038/s41467-023-44479-2)
Supplement: Supplementary file 3 — Reporting Summary [file 41467_2023_44479_MOESM3_ESM.pdf]

nature portfolio

Corresponding author(s): Raquel Lieberman and Wade van Horn

Last updated by author(s): Aug 4, 2023

Reporting Summary

Nature Portfolio wishes to improve the reproducibility of the work that we publish. This form provides structure for consistency and transparency in reporting. For further information on Nature Portfolio policies, see our Editorial Policies and the Editorial Policy Checklist.

Statistics

For all statistical analyses, confirm that the following items are present in the figure legend, table legend, main text, or Methods section.

n/a	Confirmed	
☐	☒	The exact sample size (*n*) for each experimental group/condition, given as a discrete number and unit of measurement
☐	☒	A statement on whether measurements were taken from distinct samples or whether the same sample was measured repeatedly
☒	☐	The statistical test(s) used AND whether they are one- or two-sided<br>*Only common tests should be described solely by name; describe more complex techniques in the Methods section.*
☒	☐	A description of all covariates tested
☒	☐	A description of any assumptions or corrections, such as tests of normality and adjustment for multiple comparisons
☐	☒	A full description of the statistical parameters including central tendency (e.g. means) or other basic estimates (e.g. regression coefficient) AND variation (e.g. standard deviation) or associated estimates of uncertainty (e.g. confidence intervals)
☒	☐	For null hypothesis testing, the test statistic (e.g. *F*, *t*, *r*) with confidence intervals, effect sizes, degrees of freedom and *P* value noted<br>*Give P values as exact values whenever suitable.*
☒	☐	For Bayesian analysis, information on the choice of priors and Markov chain Monte Carlo settings
☒	☐	For hierarchical and complex designs, identification of the appropriate level for tests and full reporting of outcomes
☒	☐	Estimates of effect sizes (e.g. Cohen's *d*, Pearson's *r*), indicating how they were calculated

Our web collection on statistics for biologists contains articles on many of the points above.

Software and code

Policy information about availability of computer code

Data collection	NMR data were collected with Topspin 3.6.2, X-ray data collection were conducted using SER-GUI.
Data analysis	NMR data were processed with NMRPipe and analyzed with CCPNMR AnalysisAssign V3 (v3.0.4), i-Pine server, TRENDNMR, Matlab R2022a.<br>ThT aggregation data were analyzed in Graphpad Prism 9, HDX-MS was analyzed with Protein Lynx Global Software v3.0.2 and DynamX v3.0, Crystallography software include HKL-2000, Coot, Phenix, qfit-3.0, and PyMOL.<br>PTM mass spec data were analyzed in Sequest HT within Proteome Discoverer 3.0.

For manuscripts utilizing custom algorithms or software that are central to the research but not yet described in published literature, software must be made available to editors and reviewers. We strongly encourage code deposition in a community repository (e.g. GitHub). See the Nature Portfolio guidelines for submitting code & software for further information.

Data

Policy information about availability of data

All manuscripts must include a data availability statement. This statement should provide the following information, where applicable:

- Accession codes, unique identifiers, or web links for publicly available datasets
- A description of any restrictions on data availability
- For clinical datasets or third party data, please ensure that the statement adheres to our policy

NMR data have been deposited to BMRB with ID: 51750. The crystal structure was deposited in the PDB with accession code 8FRR. The HDX-MS data have been

deposited to the ProteomeXchange Consortium with the dataset identifier PXD045520.

Research involving human participants, their data, or biological material

Policy information about studies with human participants or human data. See also policy information about sex, gender (identity/presentation), and sexual orientation and race, ethnicity and racism.

Reporting on sex and gender	NA
Reporting on race, ethnicity, or other socially relevant groupings	NA
Population characteristics	NA
Recruitment	NA
Ethics oversight	NA

Note that full information on the approval of the study protocol must also be provided in the manuscript.

Field-specific reporting

Please select the one below that is the best fit for your research. If you are not sure, read the appropriate sections before making your selection.

[x] Life sciences [ ] Behavioural & social sciences [ ] Ecological, evolutionary & environmental sciences

For a reference copy of the document with all sections, see nature.com/documents/nr-reporting-summary-flat.pdf

Life sciences study design

All studies must disclose on these points even when the disclosure is negative.

Sample size	Most experiments were conducted on at least two individually prepared protein samples except where unavailable (metal analysis, mass spectrometry). NMR data were collectively acquired on multiple different samples, often combining multiple protein purification preparations.
Data exclusions	No data were excluded from analysis.
Replication	Metal analysis was done once in technical duplicate. Replicate measurements were similar to one another.<br>For X-ray crystallography, HDX-MS, and NMR studies, single replicates were chosen as the currently established norms in these respective fields, given the time, expense, and established reproducibility. For fluorescence-based aggregation and circular dichroism-based unfolding studies, experiments were performed in triplicate which identified reported trends. Replicates included in manuscript.<br>MD simulations were conducted in triplicate for each variant. Results are generally the same and standard deviation differences are plotted along the average RMSF for each residue.<br>Mass spectrometry was conducted in analytical triplicate for each sample (1 protein prep each). All replicates were included in data analysis and plotted in the supplemental figure.
Randomization	Randomization was not used in our characterization-based observational studies. The biophysical tools used here generally give unbiased discrete outcomes. These outcomes were directly compared between mutants and other physical conditions.
Blinding	Blinding was not performed in these studies due to the resource-intensive experiments where outcomes between comparative studies are easily identifiable.

Reporting for specific materials, systems and methods

We require information from authors about some types of materials, experimental systems and methods used in many studies. Here, indicate whether each material, system or method listed is relevant to your study. If you are not sure if a list item applies to your research, read the appropriate section before selecting a response.

Materials & experimental systems

n/a	Involved in the study
☒	☐ Antibodies
☒	☐ Eukaryotic cell lines
☒	☐ Palaeontology and archaeology
☒	☐ Animals and other organisms
☒	☐ Clinical data
☒	☐ Dual use research of concern
☒	☐ Plants

Methods

n/a	Involved in the study
☒	☐ ChIP-seq
☒	☐ Flow cytometry
☒	☐ MRI-based neuroimaging
